# Superior mechanical, electrical, dielectric, and EMI shielding properties of ethylene propylene diene monomer (EPDM) based carbon black composites†

**DOI:** 10.1039/d3ra04187e

**Published:** 2023-08-24

**Authors:** Mostafizur Rahaman

**Affiliations:** a Department of Chemistry, College of Science, King Saud University P.O. Box 2455 Riyadh 11451 Saudi Arabia mrahaman@ksu.edu.sa

## Abstract

In this study, the mechanical, electrical, dielectric, and electromagnetic interference (EMI) shielding properties of ethylene propylene diene monomer (EPDM) based carbon black composites, namely high abrasion furnace (HAF) and conductive Printex blacks, were investigated and their effectiveness compared. The results show that Printex black filled composites exhibited superior properties in all aspects compared to HAF filled composites. The electrical percolation threshold value of Printex black filled composites was approximately 1/2 to 1/3 lower compared to HAF black filled composites based on classical theory and the Sigmoidal model. Moreover, the tensile modulus, dielectric permittivity, and EMI shielding efficiency (SE) of the Printex black filled composites were 4.6 times, in the order of 10^6^ at 1 kHz, and 6.65 times improved compared to HAF black filled composites at their 40 phr loadings, respectively. The Printex black filled 40 phr loaded composite showed an EMI SE of 49.94 dB that is 99.999% the attenuation of EM radiation. These properties can be attributed to the high structure of Printex black, which facilitates the ease of formation of the conductive channel through the polymer matrix, higher reinforcement, higher interfacial polarization, and high absorption of radiation. These properties were compared with some published literature on carbon black filled composites and it was found that the results of the Printex black filled composites are highly competitive with the published work. The results show that these composites are highly effective for load bearing materials, supercapacitors, and EM radiation protection.

## Abstract 2

A novel EPDM/Printex black composite exhibits approximately 50 dB of EMI SE, which means 99.999% attenuation of radiation, and could be an effective material for EMI shielding applications.

## Graphical abstract

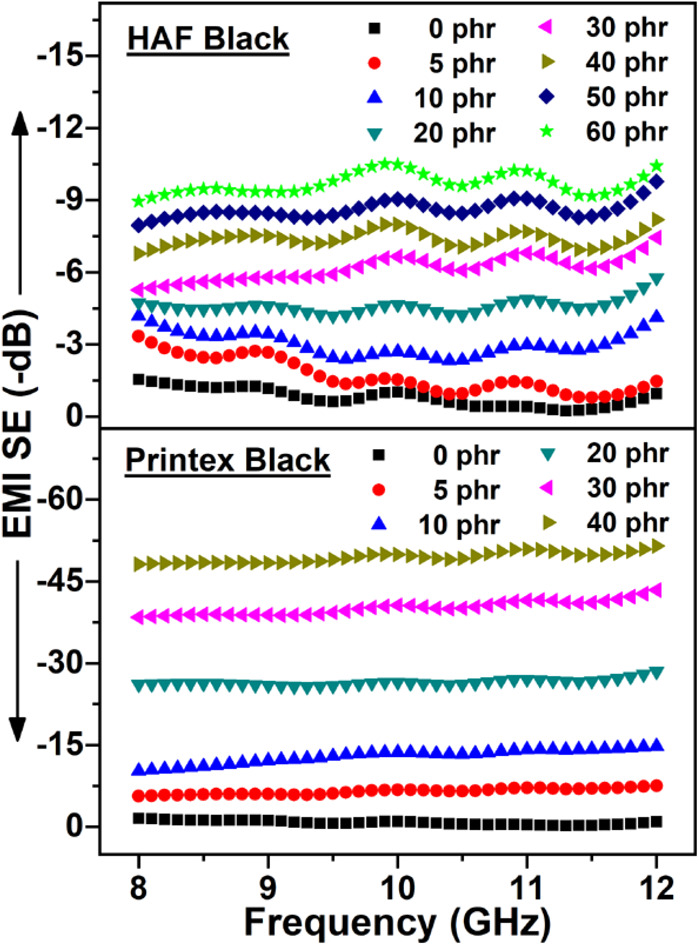

## Introduction

Most polymers are insulating because of the lack of a conjugated bonding system within their molecular structure. Hence, these polymers are not capable of conducting electricity. However, day-by-day, technology has become advanced as per the basic needs of society, environmental sustainability, and industrial requirements. One way to make these insulating polymers into electrically conductive ones is by the addition of a sufficient amount of conductive filler, which can make a channel through the polymer system and help the flow of charge carriers.^1,2^ The extrinsically conductive polymer composites prepared by this method have potential applications in electricals, dielectrics, and electronics.^3–6^ Hence, nowadays, the requirements of these types of polymer composites have increased significantly.

The most widely used and easy method of obtaining electrically conductive polymeric material is through the addition of particulate or fibrous conductive fillers into a polymer matrix.^7–9^ The addition of conductive fillers into a polymer matrix is the least expensive way of manufacturing conductive composites when compared to complicated chemical synthesis. But, this method requires the addition of a very high quantity of conductive fillers, such as conductive carbon black, to attain adequate conductivity or a low percolation threshold value.^10–12^

The electrical and dielectric properties of polypropylene (PP) based carbon black composite were investigated by Zois *et al.*^13^ The electrical conductivity was increased with an increase in carbon black loading within the polymer matrix and the percolation threshold value of conductivity was obtained at 6.2 wt% loading of carbon black. The electrical conductivity showed PTC behavior. The effect of carbon black structure on the percolation threshold value has been studied in the past.^8,14^ It is reported that the conductive carbon black has a higher structure compared to commonly used ordinary carbon black and the former gives better electrical conductivity compared to other types of carbon black at lower concentration.^8^ As a conductive filler in polymers, carbon nanotubes (CNTs) are quite effective, primarily due to their large aspect ratio.^15^ The electrical percolation threshold was reported at 0.0025 wt% of CNT loading, where the highest conductivity was 2.0 S m^−1^ at 1.0 wt% of CNT loading in epoxy matrices.^16^ Vapor grown carbon nanofiber (VGCNF) is also an effective conductive filler because of its low cost compared to CNTs.^17^ Moreover, there are many reports of using graphene as a conductive filler to modify the electrical conductivity of insulating polymers.^3,18–20^ The use of intrinsically conducting polymers such as polypyrrole or polyaniline has also provided alternative materials for the promotion of electrical conductivity in polymer-based compounds.^21,22^

The dielectric properties of polymer–carbon based composites have been studied by several authors.^5,23–25^ It has been shown that the dielectric constant and loss values increase with an increase in carbon loading but decrease with an increase in the frequency of the electric field.^26^ There are also many reports on the study of the dielectric properties of other polymer-based composites.^6,27,28^

The mechanical properties of polymer composite systems are very important from an application point of view. Ghosh *et al.* reported the tensile properties of EPDM/carbon black composites.^29^ They showed a gradual increase in tensile strength up to the highest (60 phr) loading of carbon black. The elongation at break also increased up to 25 phr of carbon black loading and then declined after further loading of carbon black. In another study, H. Ismail and co-workers investigated the tensile properties of polypropylene–natural rubber–recycle rubber powder (PP–NR–RRP)/carbon black composites.^30^ They reported that the carbon black acts as a reinforcing material, where an increase in tensile modulus and tensile strength were observed up to the highest loading of carbon black (30 wt%) but the elongation at break increased up to 10 wt% loading of carbon black and then declined. The tensile properties of polymer/carbon composite are also greatly dependent on the structure of carbon black.^31^

The EMI shielding efficiencies (SEs) of carbon-based polymer composites have also been reported in several literature studies.^9,10,32,33^ Al-Saleh *et al.* reported the EMI SE of polypropylene/high structure carbon black (PP/HSCB) composite within the X-band frequency region.^34^ They showed that the EMI SE of 17.3 dB was observed at a carbon black loading of 7.5 vol% for a 1 mm-thick film. A melt mixing process was used to prepare the composites. There are also other reports on EMI shielding materials based on polymer/carbon black composites.^10,11,35–37^

The improvement in the electrical conductivity of polymer composites is a requirement in polymer research, keeping in mind that the materials should also be economical, show good performance, and be environmentally sustainable. Conductive carbon black mixed polymer composites can be a good choice for this purpose as they exhibit a percolation threshold value at low loading. Conductive carbon black is a costly material compared to commonly used carbon blacks. But, the advantage of using conductive carbon black is that only a low quantity is required to achieve a synergistic effect with the polymer without compromising the other properties. Hence, the objective of the present study was to improve the mechanical, electrical, dielectric, and radiation shielding properties of EPDM polymer based composites filled with conductive Printex XE2 and normal HAF carbon blacks, and compare the synergistic effect of conductive Printex black over conventional HAF black when used in the same polymer matrix. The testing of tensile and tear properties, DC and AC electrical conductivity, dielectric constant and loss, and EMI shielding effectiveness was performed. The morphology of some selective samples was also checked. It was observed that the Printex black filled composites show many fold synergistic effect over HAF black filled composites. Moreover, the results of this study were compared with the results in the published literature. The novel EPDM/Printex black composite exhibited a 1071% improvement in tensile strength, 10^7^ to 10^9^-fold increment in dielectric constant value at 1 kHz frequency, and 99.999% attenuation in radiation, comparatively higher than most carbon black based composites, indicating that it could be an effective material for load bearing, supercapacitor, and EMI shielding applications.

## Materials, methods, and experimental

### Materials

Ethylene propylene diene monomer (EPDM, EP96, ML_(1+4)_ at 125 °C is 61, ethylene content is 66% ENB5, Yangtse Petrochemical, Nanjing, China) rubber was used as a matrix polymer. Two carbon blacks, namely high abrasion furnace (HAF, N330, Philips Carbon Black Limited, Durgapur, India) and Printex XE2 (Degussa Canada Limited, Burlington, ON, Canada), were used as conductive fillers. The general specifications of both carbon blacks are given in Table S1 (ESI†). Dicumyl peroxide (DCP, MP = 80 °C, purity 98%) was used as a curing agent, procured from Sigma-Aldrich chemical company (St. Louis, MO, USA). Triallyl cyanurate (TAC) was used as a co-agent for curing and supplied by E. Merck India limited (Mumbai, India). The antioxidant 1,2-dihydro 2,2,4-trimethyl quinoline (TQ, polymerized) was purchased from Lanxess India Private Limited (Bharuch, India).

### Methods

**Scheme 1:**
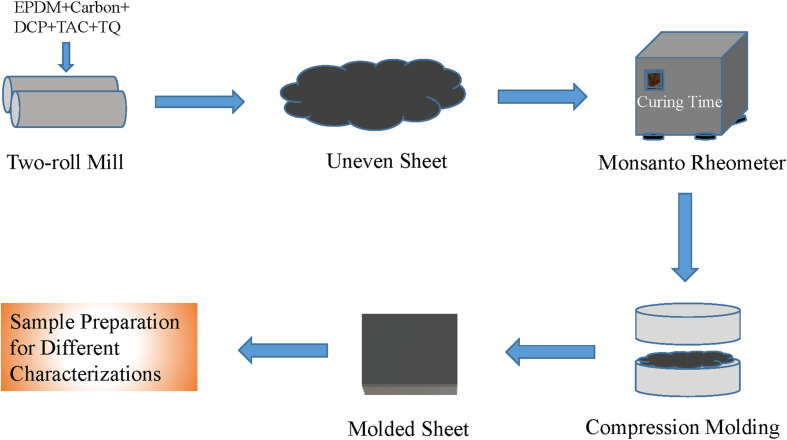
A schematic diagram for the composite preparation technique using a two-roll mixing mill.

The EPDM/carbon black composites were prepared by varying the carbon loading. All the mixings were carried out in an open two-roll mixing mill. The other ingredients, DCP, TAC, and TQ, were added sequentially as per the formulation given in Table 1. Mixing was completed within 15 min. The cure time of the mixed compound was calculated from the cure cycle graph obtained using a Monsanto rheometer R100 instrument (TA Instruments, New Castle, DE, USA). Composites were then cured in an electrically heated compression molding machine at a temperature of 160 °C and pressure of 5 MPa. The composite samples were then used in different characterization and test methods. A diagram for the composite preparation method is given in Scheme 1.

**Table 1:** Formulation of the compounding (composition parts by weight per hundred parts of rubber, phr)

Units	Ingredients					
	EPDM	HAF	PCB	DCP	TAC	TQ
phr	100	0, 5, 10, 20, 30, 40, 50, 60	0, 5, 10, 20, 30, 40	1.5	0.5	1.0
wt%	—	0, 4.76, 9.09, 16.67, 23.08, 28.57, 33.33, 37.5	0, 4.76, 9.09, 16.67, 23.08, 28.57	1.48	0.498	0.99

### Experimental

#### Mechanical properties

Mechanical testing, such as tensile strength, elongation at break, and tensile modulus, was measured according to ASTM D-412-93 using a Hounsfield H10KS universal test machine (Hounsfield Test Equipment Ltd, Redhill, UK) at an ambient temperature. Tear strength was measured according to ASTM D-624 using the same instrument. The cross-head speed and load cell for both measurements were 500 mm min^−1^ and 1 kN, respectively. The thickness of the samples was 1.0 mm. The gauge length for the tensile test samples was 40 mm and for tear tests it was 60 mm. Accordingly, the strain rate for tensile and tear tests were 12.5 per min and 8.33 per min, respectively. Each composition was tested five times and the average result was reported for data analysis.

#### Electrical properties

The DC volume resistivity of different composites with electrical resistance within the range 10^17^–10^6^ Ω was measured using an Agilent 3339B instrument (High Resistance Meter attached with Agilent 16008B Resistivity Cell, Agilent Technologies, HP, Santa Clara, CA, USA), whereas composites with a resistance below 10^6^ Ω were measured using a GOM-802 DC milli Ohm Meter (GW Instek, Good Will Instrument Co., Ltd, New Taipei City, Taiwan). The AC resistance and other dielectric properties within a frequency range of 10–10^6^ Hz were measured using a QuadTech 7600 precision LCR meter (IET Labs Inc, NY, USA) coupled with a homemade electrode. This electrode was circular type with a diameter of 12.5 mm and thickness of 1.0 mm. The reported results are the average of five tested samples for each composition. The electrical resistivity was then converted into electrical conductivity.

#### EMI SE measurement

The EMI SE of neat EPDM and its composites was measured within the X-band frequency region of 8–12 GHz. The measurement was carried out using a Scalar Network Analyzer (HP 8757C, Hewlett Packard, Agilent Technologies, Melrose, MA, USA) attached to a sweep oscillator (HP 8350B, Hewlett Packard, Agilent Technologies, Melrose, MA, USA). The sample thickness for the tests was 1.0 mm.

#### Morphological testing

The surface morphology of only some of the selected composites was analyzed using a field emission scanning electron microscope (FESEM, Model Supra 40, Carl Zeiss SMT AG, Oberkochen, Germany). The cryo-fractured surface of the samples was gold coated before their testing using a vacuum gold-sputter machine (model SC7620, Polaron Brand, Quorum Technologies Ltd, East Sussex, UK).

The structure of carbon black particles and their distribution within the EPDM matrix were investigated using a high-resolution transmission electron microscope (HRTEM, JEM 2100, JEOL Limited, Tokyo, Japan) connected to a charge couple device (CCD) camera (Gatan, Inc., CA, USA). The samples were ultramicrotomed with a Leica Ultracut UCT (Leica Microsystems GmbH, Vienna, Austria) before testing.

## Results and discussion

### Mechanical properties

#### Stress–strain behavior

**Fig. 1:**
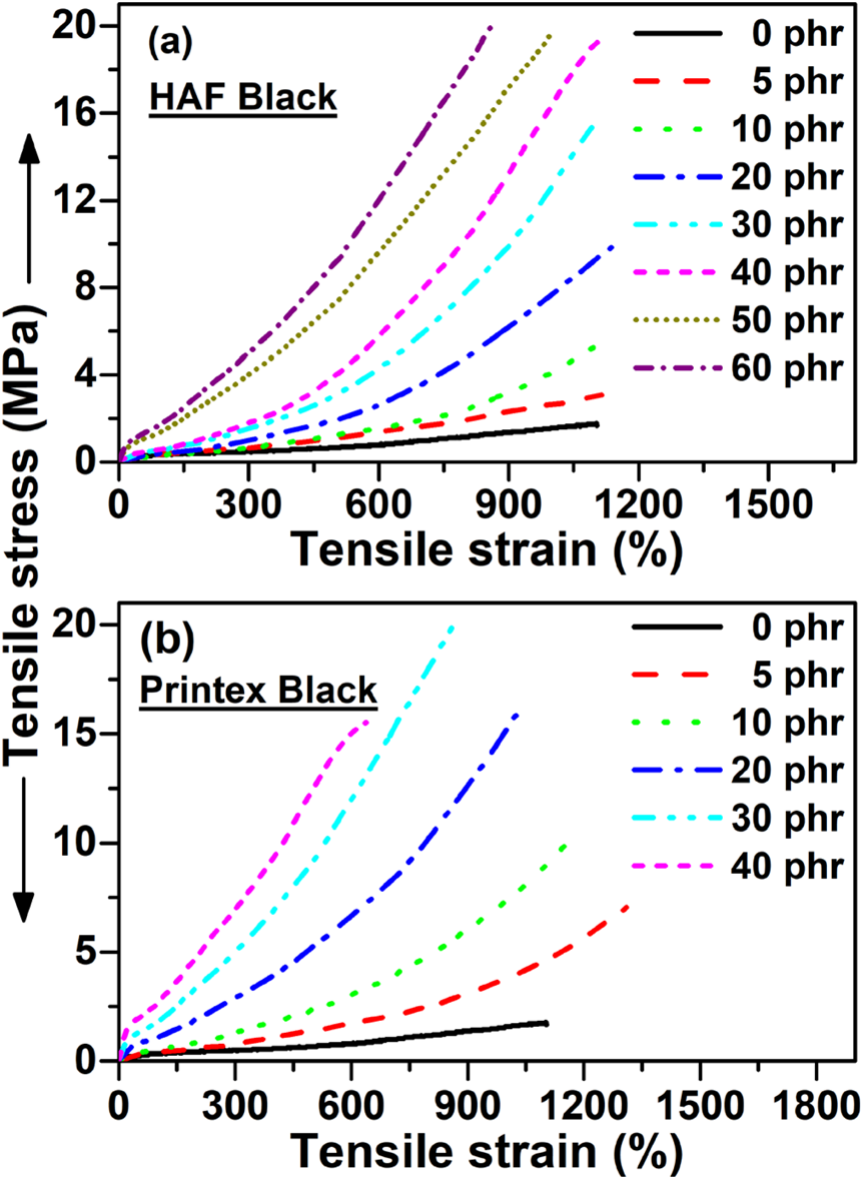
Tensile stress–strain behavior of the (a) HAF and (b) Printex black filled composites.

The tensile stress–strain behavior of the EPDM based carbon black filled composites is presented in Fig. 1. The data of tensile strength (TS), % elongation at break (% EB), and tensile modulus (TM) were extracted from the stress–strain plots and are presented in Table 2. It can be observed from the figure that the stress increases with increasing strain for all the composite systems. It can also be observed from the figure that the tensile strain at break and tensile modulus at a particular strain/elongation increases with the increase in carbon black loading for both the composites. Thus, the carbon black present in the composite system acts as a reinforcing filler for the systems. The reinforcement effect of these carbon blacks can be proved through a swelling experiment using the Kraus equation, which is given as follows (eqn (1))^9^:1
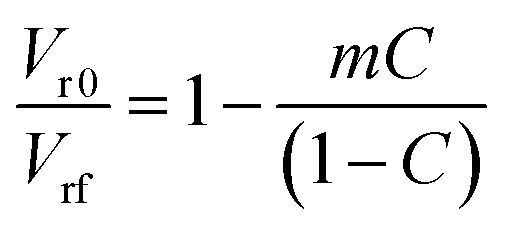
where *V*r_0_ and *V*r_f_ represent the volume fraction of neat and filled rubber in the swollen gel, respectively. *C* indicates the filler volume fraction and *m* is the polymer–filler interaction parameter, which is calculated from the slope of the plot of *V*r_0_/*V*r_f_*vs. C*/(1 − *C*). The volume fraction of rubber (*V*_r_) can be calculated using eqn (2):2
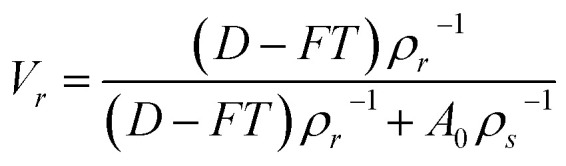
where *T*, *D*, and *A*_0_ are the weight of the test specimen, de-swollen test specimen, and absorbed solvent, respectively. *F* represents the fractional weight of the insoluble component in the sample. *ρ*_r_ and *ρ*_s_ are the densities of rubber and solvent, respectively. When plotted, *V*r_0_/*V*r_f_*vs. C*/(1 − *C*) shows a straight line with either a positive or a negative slope. The appearance of the positive slope represents a strong polymer–filler interaction and negative slope weak polymer–filler interaction.

**Table 2:** Tensile and tear strength (MPa), elongation at break (%), and modulus (MPa) @50% strain with respect to filler loading (FL, in phr)

FL	HAF						Printex					
	Tensile			Tear			Tensile			Tear		
	TS	EB	TM	TS	EB	TM	TS	EB	TM	TS	EB	TM
0	1.7 ± 0.2	1104 ± 78	0.49 ± 0.03	15.3 ± 1.2	289 ± 21	14.0 ± 1.3	1.7 ± 0.2	1104 ± 78	0.49 ± 0.03	15.3 ± 1.2	289 ± 21	14.0 ± 1.3
5	3.1 ± 0.3	1115 ± 83	0.50 ± 0.03	20.7 ± 2.3	335 ± 26	16.0 ± 1.4	7.1 ± 0.5	1311 ± 114	0.58 ± 0.04	27.7 ± 2.5	342 ± 29	18.6 ± 1.9
10	5.4 ± 0.4	1107 ± 86	0.52 ± 0.04	28.7 ± 2.7	349 ± 32	17.4 ± 1.5	10.6 ± 0.8	1185 ± 103	0.78 ± 0.06	50.0 ± 3.8	311 ± 26	26.6 ± 2.7
20	9.9 ± 0.6	1138 ± 92	0.56 ± 0.04	46.7 ± 3.4	331 ± 27	21.4 ± 1.8	15.9 ± 1.0	1025 ± 69	1.56 ± 0.11	78.0 ± 5.4	222 ± 22	50.6 ± 4.3
30	15.8 ± 0.8	1108 ± 81	0.80 ± 0.05	61.7 ± 4.2	289 ± 19	26.0 ± 2.2	19.9 ± 1.3	860 ± 56	2.64 ± 0.23	103.0 ± 7.8	161 ± 18	92.8 ± 6.6
40	19.3 ± 1.2	1105 ± 75	0.86 ± 0.06	63.3 ± 4.6	260 ± 17	29.4 ± 2.5	15.5 ± 1.1	638 ± 48	3.98 ± 0.34	97.0 ± 6.4	94 ± 8	134.0 ± 8.4
50	19.8 ± 1.3	1005 ± 63	1.98 ± 0.12	62.2 ± 4.5	183 ± 14	37.4 ± 3.1	—	—	—	—	—	—
60	20.1 ± 1.3	862 ± 52	2.48 ± 0.21	59.3 ± 4.3	141 ± 12	47.2 ± 3.9	—	—	—	—	—	—

**Fig. 2:**
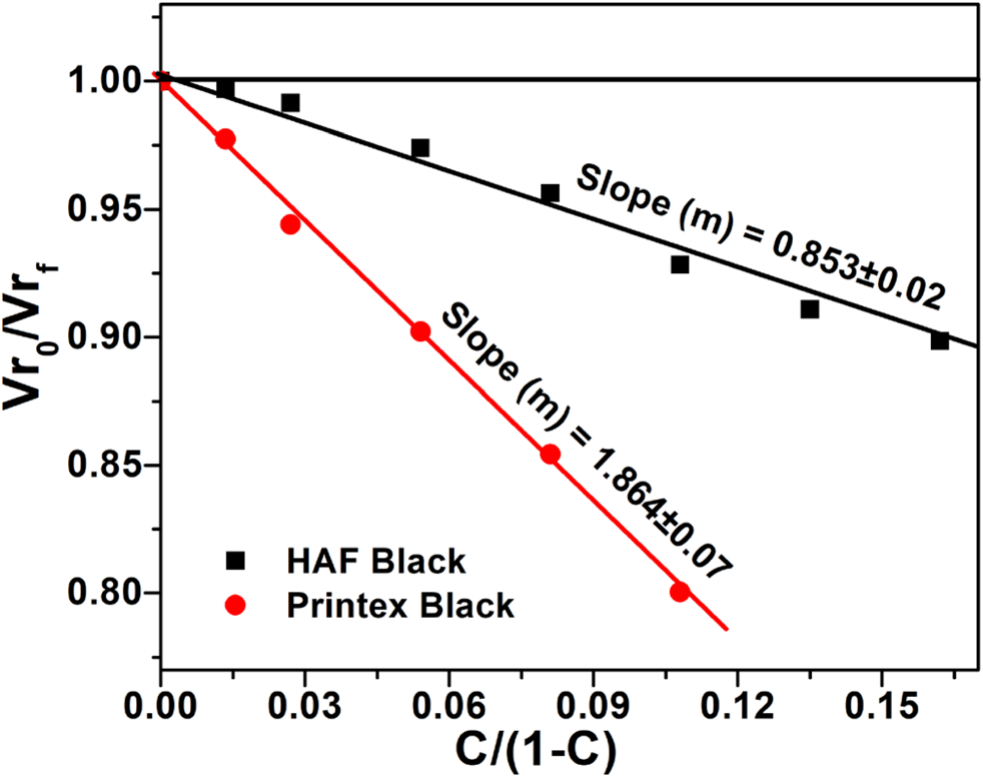
Kraus plots of HAF and Printex black filled composites.

The slope of *V*r_0_/*V*r_f_*vs. C*/(1 − *C*) will be positive for reinforcing filler; whereas, for non-reinforcing filler, the slope will be negative. The plots based on the Kraus equation are shown in Fig. 2, where the plots exhibits that the slopes are positive for both carbon black-filled systems. Thus, it can be said that both carbon blacks act as reinforcing fillers for the composites. It is also revealed from the figure that the slope value is more positive for the Printex black filled composite compared to the HAF black filled one. Thus, the effect of reinforcement of Printex black is better than that of HAF black. This is consistent with the experimental results from the stress–strain plots. Actually, different organic functional groups, such as –OH, –COOH, –CHO, >CO, *etc.*, are present on the surface of the carbon blacks. These groups reinforce the composite by physical bonding with the polymer matrix. This is why the tensile strength increases with the increase in filler loading. This happens for both the composite systems. However, the higher tensile strength and modulus for the Printex black filled composite at low filler loading is due to the higher structure of Printex black, which leads to better polymer–filler interaction for Printex black compared to HAF black in the polymer matrix. It can be observed from the figure and table that the elongation at break initially increases up to a certain filler loading and then decreases after further addition of carbon blacks. Initially, the increase in elongation at break is due to the reinforcing effect of carbon black, but at higher filler concentration the molecular chain flexibility is reduced, which results in the decrease of elongation at break after further addition of carbon black. The highest increment in the tensile properties is presented in Table 3. It is envisaged from the table that the highest increase in all the tensile properties is observed at a lower filler loading for the Printex black filled composites. Hence, Printex black is more effective than HAF black for improving tensile properties. The tensile strength (TS) results in this study were compared with some published literature studies, as shown in Table 4. In this table, the composite preparation methods, the TS of pure polymers, the highest increment in TS at the particular filler loading, and % increment in TS compared to pure polymers are reported. It can be observed from the table that the TS of the base EPDM polymer is substantially lower than the base polymers. Moreover, the highest TS values of most of the composites are also high compared to the EPDM composite but the % increment is the highest among all the other composites mentioned herein. Hence, again, it can be inferred that the Printex black is more effective than the other many blacks in improving tensile strength.

**Table 3:** Highest increment in properties with respect to filler loading (FL)

Properties	HAF				Printex			
	Tensile		Tear		Tensile		Tear	
	% increase	@FL	% increase	@FL	% increase	@FL	% increase	@FL
TS	1082	60	314	40	1071	30	573	30
EB	3	20	21	10	19	5	18	5
TM	406	60	237	60	712	40	857	40

**Table 4:** Tensile strength (TS) value of different carbon blacks and carbon black based hybrid polymer composites (in some cases, approximate values are given)

Composites^a^	Preparation method	TS of base polymer (MPa)	Highest TS of composite (MPa)	% increment	FL at the highest TS (phr/wt%)	Ref.
EMA/CB	Melt blending	8.0	12.2	52.5	40 wt%	38
CPE/CB	Melt blending	7.6	30.8	305	30 wt%	39
EMA/EOC/CB	Solution mixing	8.3	16.3	96	15 wt%	40
ABS/CB	Melt compounding	45.1	47.5	5	3 wt%	41
CPE/CNF/CB	Solution blending	7.6	28.2	271	(7.5 + 7.5) wt%	42
UHMWPE/PP/CB	Mechanical mixing	23.45	31.1	32.6	3 phr	43
PI/CB	Solution mixing	100	297	197	10 wt%	44
SS/HDPE/CB	Melt extrusion	3.8	9.3	144.7	15 wt%	45
NBR/CB	Melt mixing	3.9	17.2	341	25 wt%	46
TPU/PLA/CB	Solution mixing	16.1	40	148.4	30 wt%	47
EPDM/PCB	Two-roll mixing	1.7	19.9	1071	23.08 wt%/30 phr	This work

a Abbreviations: EMA = ethylene methyl acrylate, CB = carbon black, CPE = chlorinated polyethylene, EOC = ethylene octane copolymer, ABS = acrylonitrile butadiene styrene, CNF = carbon nanofiber, UHMWPE = ultra-high molecular weight polyethylene, PP = polyethylene, PI = polyimide, SS = sorghum straw, HDPE = high density polyethylene, NBR = acrylonitrile butadiene rubber, TPU = thermoplastic polyurethane, PLA = polylactic acid, and PCB = Printex carbon black.

**Fig. 3:**
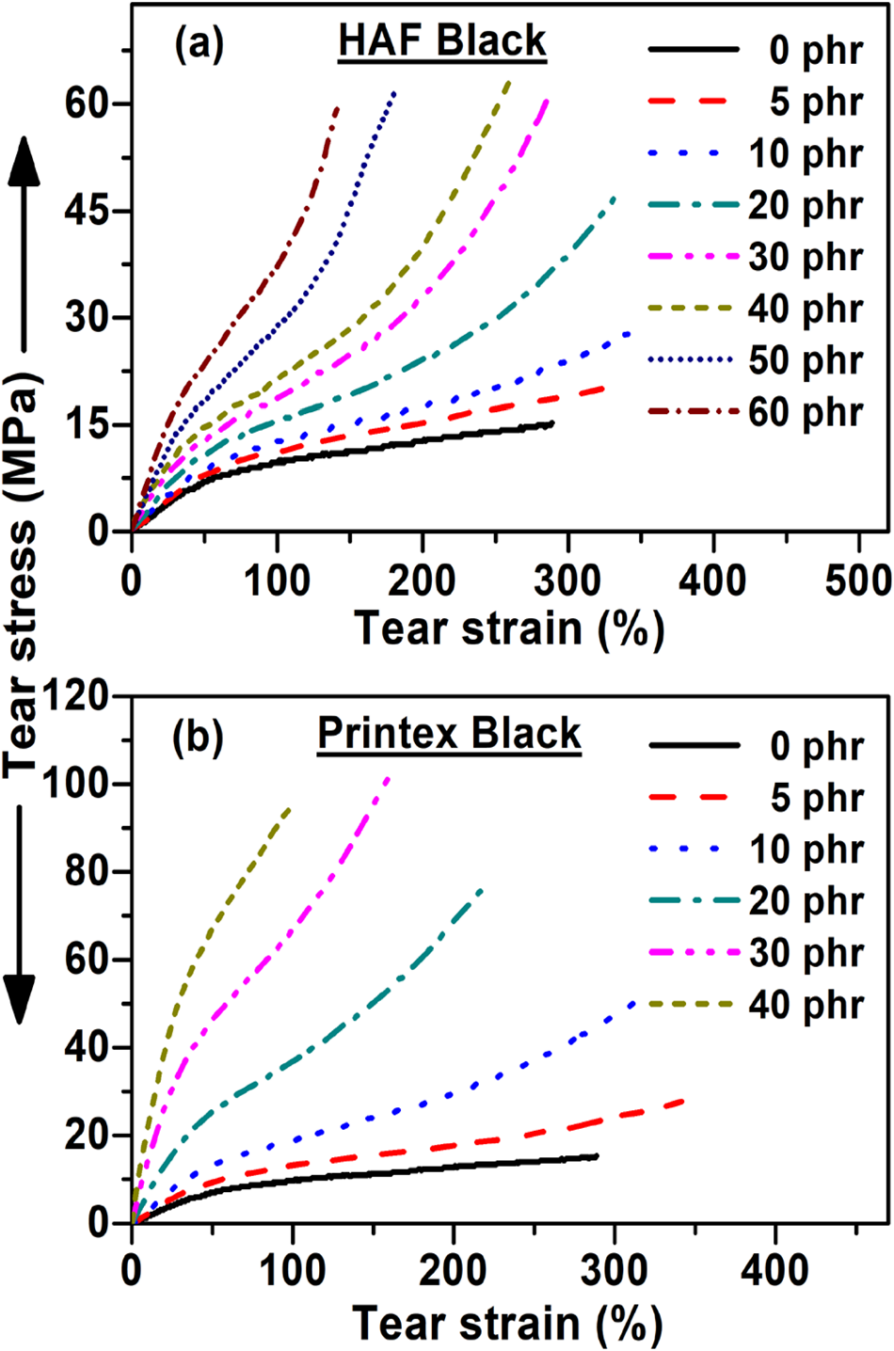
Tear stress–strain behavior of the (a) HAF and (b) Printex black filled composites.

The tear stress–strain behavior of the composites is presented in Fig. 3. It can be observed from this figure that the tearing stress–strain behavior is similar to the tensile stress–strain behavior. The only difference arises in the magnitude of their parameter value, where the tear strength and modulus values are higher compared to the tensile strength and modulus values, as shown in Table 2. On the contrary, the value of the tensile EB is higher than the tear EB value. From Table 3, it can be observed that the similar tensile properties and tear properties of the Printex black filled polymer composites lead to them exhibiting the best properties at low filler loadings.

#### DC conductivity

**Fig. 4:**
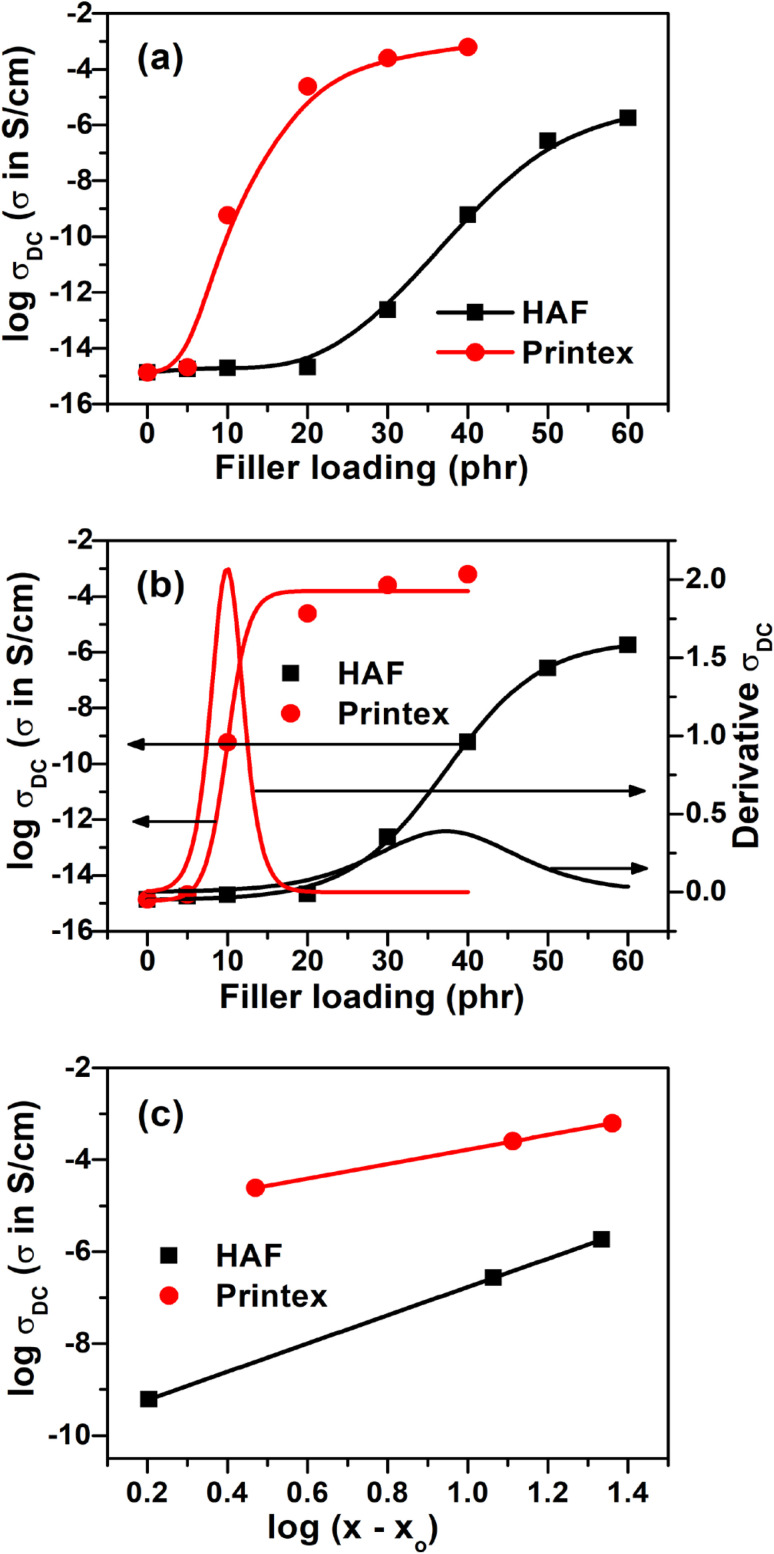
(a) DC conductivity *vs.* filler loadings of the HAF and Printex black filled composites, (b) plots based on the Sigmoidal-Boltzmann model, and (c) plots based on classical percolation theory.

The variation in DC conductivity against filler loading for EPDM composites filled with two different carbon fillers, namely high-abrasion furnace black (HAF) and Printex black, is presented in Fig. 4. It can be observed from the figure that initially the DC conductivity increases marginally for the HAF black filled composite up to a certain filler loading (20 phr) and thereafter a sudden increase in conductivity is observed within the filler loading range of 30–40 phr followed by a marginal change in conductivity up to the highest filler loading. However, for the Printex black filled EPDM composites, initially, the increase in DC conductivity is quite slow up to 5 phr loading and then a sharp increase in conductivity is observed at a relatively lower filler loading that is around 10 phr compared to the HAF black filled composites. Finally, the change in conductivity becomes marginal after further addition of Printex black. The percolation threshold (critical concentration) for the Printex black filled composites was found to be lower compared to the HAF black filled composites, as calculated from the Sigmoidal–Boltzmann model, which is given as follows:^14^3
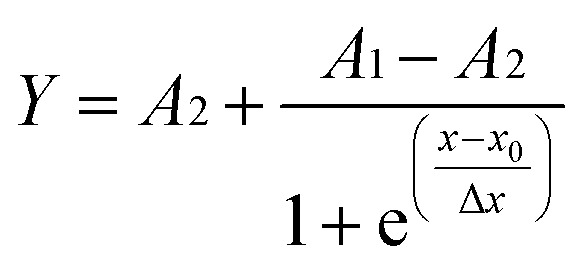
where *Y* is the conductivity of the composite at the corresponding filler loading *x*, *A*_1_ is the conductivity of filler, *A*_2_ is the conductivity of the polymer, *x*_0_ is the percolation threshold and Δ*x* is a fitting parameter. The percolation threshold for the HAF black filled composite was 37.3 phr (27.17 wt%), whereas for Printex black filled composite, it was 9.9 phr (8.27 wt%). Actually, the electrical conductivity and percolation threshold for any extrinsically conducting composite depends on the nature of the polymer matrix, the nature of the filler, the crystallinity in the polymer, the viscosity of the polymer matrix, the structure of the carbon filler, *etc.* Herein, we used single-matrix EPDM, which is amorphous and flexible in nature. Hence, the electrical conductivity and percolation threshold fully depend on the nature of the filler, that is, the structure of the carbon fillers. The two fillers used, namely HAF and Printex black, differ largely in their structure (aggregating tendency). The DBP (dibutyl phthalate) absorption value of HAF black is 88–102 cc/100 g, whereas for Printex black it is 350–410 cc/100 g, as shown in Table S1 (ESI†). Hence, the structure of Printex black is approximately 4 times higher compared to HAF black (the structure of carbon black is measured in terms of DBP (dibutyl phthalate) absorption value). The higher the structure of carbon black, the lower its percolation threshold and the higher its electrical conductivity. In fact, a higher structure facilitates the formation of a continuous conducting network easily at a lower filler loading. This is why the composite filled with Printex black exhibits higher conductivity and a lower percolation threshold compared to the HAF black filled composite. In the case of the Printex black filled composite, the percolation threshold value is approximately 4 times lower than that of the HAF black filled composite. Hence, we observed an almost inverse relationship in the difference of the structure of the carbon blacks and their percolation thresholds.

The percolation threshold of electrical conductivity was also determined by classical percolation law, which is as follows:^22^

4 *σ* = *σ*_o_(*x* − *x*_o_)^*t*^ for *x* > *x*_o_

where *σ* is the conductivity of the composites, *t* is the critical exponent, *x* is the wt% of the filler, *x*_o_ is the wt% at the critical concentration/percolation threshold, and *σ*_o_ is a constant, indicating the conductivity at the percolation threshold. Taking logarithm of both sides of eqn (4) gives log *σ* = log *σ*_o_ + *t* × log(*x − x*_o_). Plotting log *σ* against log(*x* − *x*_o_), we have a straight line with a slope *t* and intercept log *σ*_o_. The value of *t* can be obtained from the slope and the value of *σ*_o_ can be obtained from the intercept. For a three-dimensional system, the value of *t* is in the range of 1.65–2. The equation of the best fit (at the coefficient of correlation, *R*^2^ = 1) is obtained at the filler loading 38.4 phr (27.75 wt%) for the HAF black and at a loading of 17.05 phr (14.57 wt%) for the Printex black filled composites. Hence, the percolation threshold value for the EPDM/HAF black composite is 38.4 phr (27.75 wt%) and for the EPDM/Printex black composite it is 17.05 phr (14.57 wt%). It was observed that the values of the percolation thresholds determined by classical percolation theory are quite high compared to the percolation thresholds values determined using the Sigmoidal-Boltzmann model when considering a similar composite system. A similar type of observation was made in our earlier study.^48^ This discrepancy arises because of the assumption of different theoretical concepts. In the Sigmoidal-Boltzmann model, we considered all the compositions to determine the percolation threshold value and the maxima of the derivative plots gives the percolation threshold value, whereas in the case of the classical percolation theory, we are assuming only compositions that have conductivity above the percolation threshold and are exponential in nature. As a result, during calculation, the percolation threshold value is shifted to a higher value. The critical exponent, *t*, obtained from the slope of the plots is 3.07 and 1.58 for the HAF and Printex black filled composites, respectively. Hence, this *t* value for EPDM/HAF composites is high but for EPDM/Printex composites is low compared to a universal three-dimensional system.^49^ This type of discrepancy has also been reported in previous studies.^22,50^ The rate of increase in conductivity beyond the percolation threshold is responsible for this type of discrepancy. A high rate of increase gives a high *t* value. The log *σ*_o_ values for the EPDM/HAF and EPDM/Printex composites are −9.84 and −5.35, respectively. Hence, the conductivities, *σ*_o_, at the percolation threshold are 1.4 × 10^−10^ S cm^−1^ and 4.4 × 10^−6^ S cm^−1^ for the EPDM/HAF and EPDM/Printex composites, respectively. This value of electrical conductivity is quite low compared to the electrical conductivity of carbon black alone. It can be attributed that the carbon particles are coated with the insulating polymer layers within the polymer composite systems, which results in poor contact between the carbon particles and the effective result of contact resistance rather than the resistance of individual carbons.

The DC conductivity and percolation threshold values from previously published literature are shown in Table 5 and compared with the results of this study. It can be observed from the table that some hybrid, ternary, and crystalline polymer based composites exhibit a low percolation threshold and high electrical conductivity as a result of the selective localization of filler particles within the polymer matrix that form conductive pathways at low filler loading. The SR/CB and SR/GN composites exhibited comparatively low electrical conductivities at their highest filler loadings but also exhibited low percolation threshold values. The conductivity and percolation threshold value of some carbon black based composites are similar to the present study but in many cases low conductivity and high percolation threshold values are observed. Though Printex black is an effective filler, the discrepancy in results arises because there are differences in the conductivity value of the base polymers or their blends.

**Table 5:** Conductivity of different carbon blacks and carbon black based hybrid polymer composites (AC conductivity at 1 kHz, in some cases approximate values are given)

Composites^a^	Filler loading (phr/wt%/vol%)	Preparation method	Conductivity (S cm^−1^)			Ref.
			DC	AC	*x*_o_	
PP/CB	10 vol%	Melt blending	1.0 × 10^−1^	—	1–3	34
PP/PA6/CB	25 wt%	Melt compounding	2.3 × 10^−1^	—	10–15	52
PP/PA6/CB/SF	(25 + 25) = 50 wt%		8.6 × 10^−1^	—	10–20	
PS/EVA/CB	15 wt%	Melt blending	2.4 × 10^−1^	1.0 × 10^−3^	3–6	53
EMA/CB	40 wt%	Melt blending	6.7 × 10^−4^	8.9 × 10^−4^	18.1	38
CPE/CB	40 wt%	Melt blending	2.4 × 10^−3^	8.8 × 10^−4^	13.7	39
PU/CB	24 phr	Solution casting	—	3.2 × 10^−3^	—	54
PU/GN			—	0.8 × 10^−3^	—	
EMA/CB	30 wt%	Solution mixing	9.6 × 10^−5^	8.7 × 10^−6^	20	40
EOC/CB			4.5 × 10^−5^	6.4 × 10^−6^	20	
EMA/EOC/CB			1.0 × 10^−2^	8.6 × 10^−5^	10	
SR/CB	8 wt%	Solution mixing	0.3 × 10^−4^	2.2 × 10^−4^	1	55
SR/GN			9.8 × 10^−6^	9.2 × 10^−6^	2	
PA6,6/CB	30 wt%	Melt extrusion	5.2 × 10^−2^	—	15–20	56
PVDF/CB	7.5 wt%	Dry blending followed by melt mixing	—	7.0 × 10^−4^	—	57
PVDF/EG	15 wt%		—	6.6 × 10^−4^	—	
PVDF/CB/EG	(7.5 + 2) = 9.5 wt%		—	7.0 × 10^−3^	—	
PP/CB	30 wt%	Steam-chest molding	1.3 × 10^−2^	—	15	58
PP/CNT/CB	(2.5 + 2.5) = 5 wt%	Mechanical mixing	6.2 × 10^−2^	—	0.47 vol%	59
CPE/CNF/CB	(7.5 + 7.5) = 15 wt%	Solution blending	2.2 × 10^−2^	1.4 × 10^−2^	4.8	42
PVDF/CB/Fe_3_O_4_	(30 + 10) = 40 wt%	Solution mixing and coagulation process	6.4 × 10^−2^	—	—	60
PMMA/CB	10 wt%	Solution mixing	1.2 × 10^−3^	1.0 × 10^−3^	2.79	61
UHMWPE/PP/CB	15 phr	Mechanical mixing	1.1 × 10^−1^	—	0.48	43
PI/CB	40 wt%	Solution mixing	5.2 × 10^−1^	—	15–20	44
SS/HDPE/CB	25 wt%	Melt extrusion	0.4	—	—	45
TPU/PLA/CB	30 wt%	Solution mixing	1.2 × 10^−4^	—	20	47
EPDM/PCB	16.67 wt%/20 phr	Two-roll mixing mill	2.5 × 10^−5^	5.0 × 10^−3^	8.27 wt%/9.9 phr	This work
	23.08 wt%/30 phr		2.5 × 10^−4^	2.5 × 10^−2^		
	28.57 wt%/40 phr		6.3 × 10^−4^	3.2 × 10^−2^		

a Abbreviations: PA6 = polyamide 6, PS = polystyrene, SF = sisal fiber, EVA = ethylene vinyl acetate, PU = polyurethane, GN = graphite nanoparticle, PVDF = polyvinyl difluoride, CI = carbonyl iron, SR = silicone rubber, PA6,6 = polyamide 6,6, MWCNT = multiwalled carbon nanotubes, EG = expanded graphite, PMMA = polymethyl methacrylate, PDMS = polydimethyl siloxane, and SBR = styrene butadiene rubber.

#### AC conductivity

**Fig. 5:**
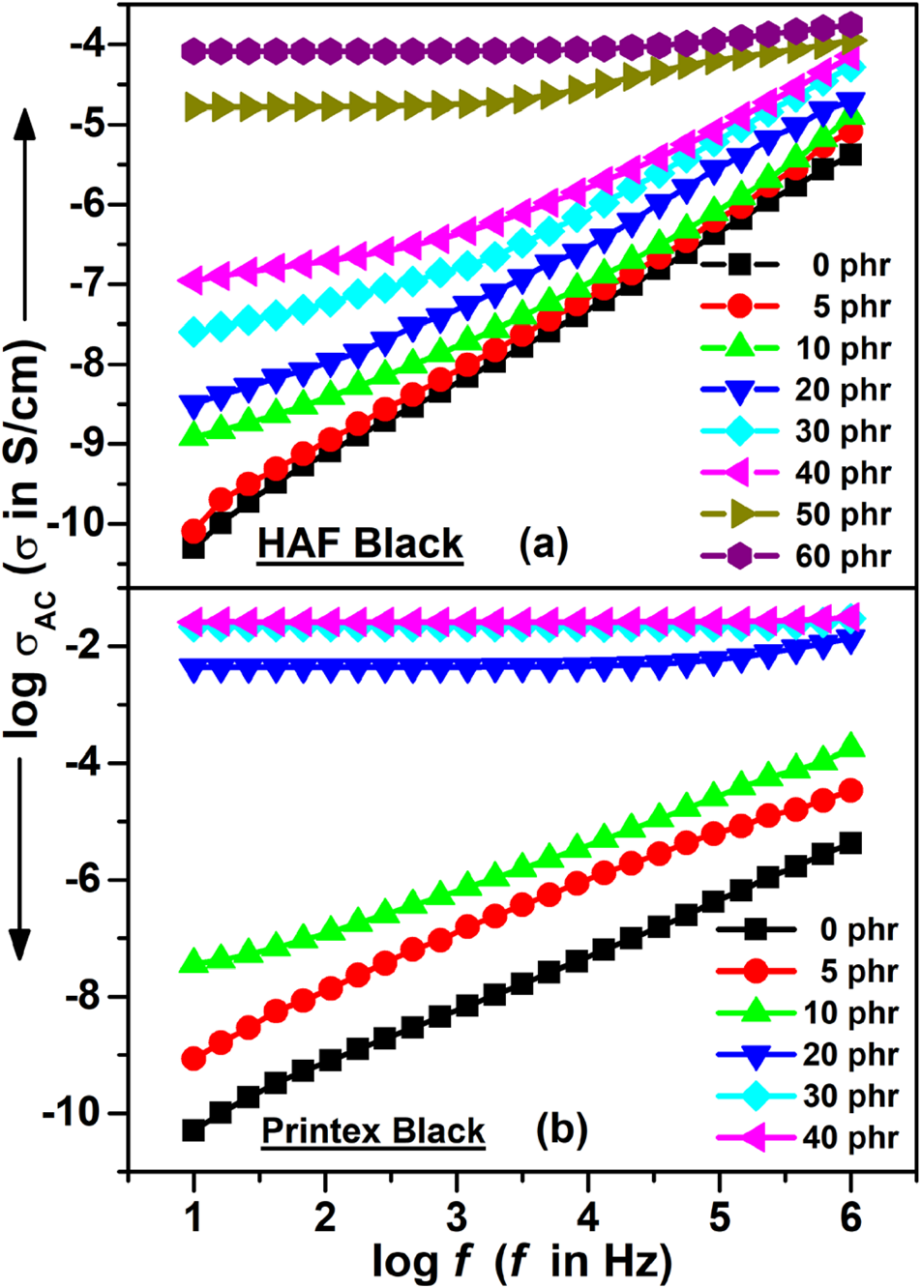
AC conductivity *vs.* frequency for the (a) HAF and (b) Printex black filled composites.

The variation in AC conductivity against frequency for the EPDM composites filled with HAF and Printex blacks is shown in Fig. 5a and b, respectively. It can be observed from the figures that the composites with a filler loading below the percolation threshold exhibit frequency dependent behavior, where the AC conductivity increases with an increase in frequency. In contrast, the composites with a filler loading beyond the percolation threshold exhibit near frequency independent behavior. The increase in frequency results in an increase in the activation energy of the electron present in the system. This results in an increase in the hopping/tunneling of the electrons. This hopping/tunneling of electrons increases the AC conductivity for any composite. This is why the AC conductivity increases with increasing frequency for the composites with a filler loading below the percolation threshold, whereas for a composite with a filler loading above the percolation threshold there is the formation of a continuous conducting network. Hence, in this case, the electrons present in the system do not need to hop through the system for current to flow because there is particle–particle contact. This is why these composites exhibit near frequency independent behavior. Herein, the conductivity is entirely due to the flow of DC current. The composites with a filler loading of around the percolation threshold exhibit both frequency independent and dependent behavior. Hence, there is an onset/critical frequency below which the composite exhibits frequency independent behavior and above which frequency dependent behavior. Frequency independent behavior is observed for 50 phr loaded EPDM/HAF composites from a frequency of 10 Hz to 10^3^ Hz and frequency dependent behavior is observed from a frequency of 10^3^ Hz to 10^6^ Hz. Similarly, for 60 phr loaded EPDM/HAF composites, frequency independent behavior is observed from 10 Hz to 10^4^ Hz and frequency dependent behavior from frequency 10^4^ Hz to 10^6^ Hz. On the contrary, the 20 phr loaded Printex black filled composite exhibits frequency independent behavior from 10 Hz to 10^5^ Hz and frequency dependent behavior from 10^5^ Hz to 10^6^ Hz. Hence, the characteristic onset frequency for the 50 phr loaded EPDM/HAF, 60 phr loaded EPDM/HAF, and 20 phr loaded EPDM/Printex black filled composites are 10^3^ Hz, 10^4^ Hz, and 10^5^ Hz, respectively. This indicates that this characteristic onset frequency is shifted to a higher frequency value when the filler loading is increased. Initially, the frequency independent behavior is due to the free mobile charges, which are available within the composite system, and, lastly, the dependent behavior of the conductivity is due to the trapped charges, which become active only in higher frequency regions. It was observed that 30 and 40 phr loaded Printex black filled composites exhibited almost frequency independent behavior over the entire frequency range. Kilbride and co-workers proposed the relationship between the AC conductivity at critical frequency *σ* (fc) and the DC conductivity *σ*_dc_ to be *σ* (fc) = 1.1 *σ*_dc_.^51^ Accordingly, the AC conductivities at critical frequency as per this relationship for the 50 phr loaded EPDM/HAF, 60 phr loaded EPDM/HAF, and 20 phr loaded EPDM/Printex black filled composites are −7.2250 S cm^−1^, −6.3153 S cm^−1^, and −5.0741 S cm^−1^, respectively. However, the experimental AC conductivities at the critical frequency for the 50 phr loaded EPDM/HAF, 60 phr loaded EPDM/HAF, and 20 phr loaded EPDM/Printex black filled composites are −4.7439 S cm^−1^, −4.0625 S cm^−1^, and −2.2351 S cm^−1^, respectively. Thus, there is quite a difference between the AC conductivities at critical frequency and their DC conductivities. This can be attributed to the fact that at high frequency the charge carriers become more activated and this results in a higher AC conductivity.

The AC conductivity was also found to increase with increased carbon black loading at any particular frequency. The AC conductivity for the Printex black filled composite was also found to be higher compared to the HAF black filled composite at any particular filler loading. This can be explained in the same manner as the DC conductivity increment with respect to the carbon loading. The AC electrical conductivity results of this study were compared with the published work of other researchers, as reported in Table 5. It can be observed from the table that the AC conductivity of the hybrid CPE/CNF/CB composite is only slightly higher compared to that in this study. This is because of the formation of a preferential network of hybrid fillers within the polymer matrix. Irrespective of the filler loading and processing methods, the AC conductivity results in this study are higher compared to the other composites. However, it also cannot be ignored that some of the composites exhibit good AC conductivity at a low filler loading.

#### Dielectric constant and loss

**Fig. 6:**
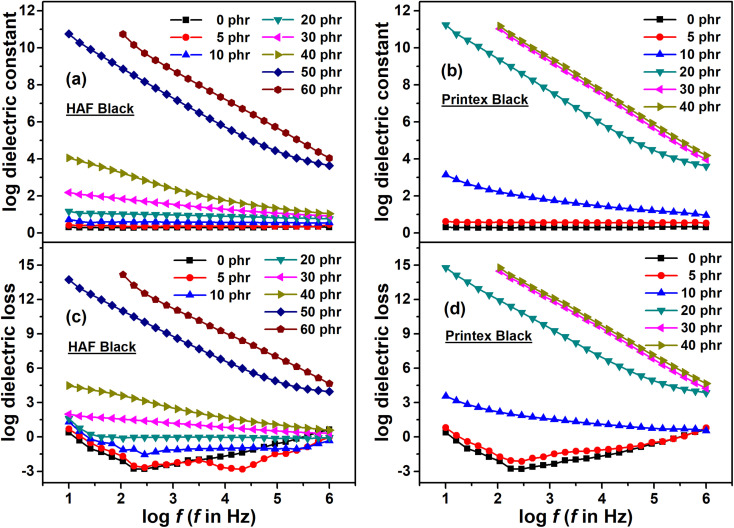
Dielectric constant *vs.* the frequency of the (a) HAF and (b) Printex black filled composites. Dielectric loss *vs.* the frequency of the (c) HAF and (d) Printex black filled composites.

The effect of frequency on dielectric constant and loss for EPDM composites filled with HAF and Printex blacks is shown in Fig. 6. It can be observed from the figure that the composites with a filler loading below the percolation threshold exhibit frequency independent behavior for both black-filled systems. However, the composites with a filler loading above the percolation threshold show frequency dependent behavior. The filler particles present in the composite system act as micro capacitors/dipoles when two filler particles are separated by a layer of insulating polymer matrix. With the application of an electric field, the dipoles present in the polymer matrix orient themselves along the direction of the applied electric field. With an increase in frequency, these dipoles have very little time to orient themselves along the direction of the applied electric field. This is why the dielectric constant and loss decrease with an increase in the frequency for a composite with a filler loading above the percolation threshold. Above the lower frequency region, the effect of interfacial polarization between the polymer matrix and filler particles contributes more to the dielectric constant and loss. However, with a gradual increase in frequency, the effect of interfacial polarization is gradually reduced, which results in a decrease in dielectric constant and loss with an increase in frequency. For a composite with a filler loading below the percolation threshold, the particles present in the composite are apart from each other, and hence the effect and generation of interfacial polarization is less in number and magnitude. This is why these composites exhibit frequency independent behavior.

It is also observed from the figures that with an increase in filler loading for both composite systems the dielectric constant and loss increased. The base polymer matrix EPDM is non-polar in nature, hence, its contribution to the increase in dielectric properties (dielectric constant and loss) is insignificant. Actually, the dielectric properties are the net result of different polarization effects, namely electronic polarization, atomic or group polarization, and interfacial polarization arises when an AC electric field is applied. The most important property that contributes more to the increase in dielectric properties is the interfacial polarization that originates at the interface of the polymer matrix and filler particles. It was mentioned earlier that the filler particles present in the composite system act as micro capacitors or dipoles. With an increase in filler loading, the number of such micro capacitors or dipoles increases, which in turn increase the effect of interfacial polarization. This increase in interfacial polarization results in an increase in capacitance, which finally leads to an increase in dielectric constant and loss.

If we carefully look at the figure, it can be observed that the composite filled with Printex black exhibits a higher dielectric constant and loss compared to the HAF black filled composite when considering equal filler loading at any particular frequency. It was mentioned earlier that the filler particles present in the composite system act as a micro capacitor/dipole. Thus, a carbon black particle with a higher structure may be considered to be a combination of two or more capacitors of carbon black with a lower structure. Herein, the Printex black has a higher structure compared to that of HAF black. The DBP absorption value of Printex black is 350–410 cc/100 g, whereas for HAF black it is 88–102 cc/100 g. Thus, the structure of Printex black is 3–4 times greater than that of HAF black. Hence, the capacitor made from Printex black is equivalent to a capacitor made from 3–4 HAF black particles. This is why Printex black filled composite exhibits a higher dielectric constant and loss compared to the composites made from HAF black at equal filler loading at any particular frequency. If we consider the dielectric constant value of 40 phr loaded composites at a particular frequency of 1 kHz, it can be observed that the increment in the dielectric constants for the HAF and Printex black filled composites are in the order of 10^3^ and 10^9^, respectively. Though the increments in both cases are high, the Printex black filled composite shows an extremely high value compared to the HAF composite at an equal filler loading. However, the 60 phr loaded HAF filled composite shows an improvement in dielectric constant in the order of 10^9^. Many authors have also studied the dielectric properties of polymer–carbon composites. The dielectric constant values from some published literature studies are presented in Table 6. The dielectric constant data are noted at a frequency of 1 kHz. It can be observed from the table that the hybrid epoxy/CB/HGB/rGO composite exhibits a good dielectric constant value at a very low filler loading. The rest of the composites are mostly carbon black based composites. However, the improvement in the dielectric constant values of these composites are less effective compared to in this study, as can be seen from the Table 6. In this study, the level of improvement is in the order of 10^7^ to 10^9^. This level of improvement in the dielectric constant value indicates that these composites can be used as supercapacitors.

**Table 6:** Dielectric constant values of different carbon blacks and carbon black based hybrid polymer composites at 1 kHz

Composites^a^	Preparation method	Filler loading (phr/wt%/vol%)	Dielectric constant value	Ref.
PU/CB	Solution casting	24 phr	∼4 × 10^3^	54
PU/GN			∼1.2 × 10^3^	
SR/CB	Solution mixing	8 wt%	∼8 × 10^3^	55
SR/GN			∼1.1 × 10^2^	
Epoxy/CB	Brabender kneader	7 vol%	∼2.2 × 10^3^	62
AEM/CB	Two-roll mixing	30 phr	∼4 × 10^4^	63
Epoxy/CB	Mechanical mixing	3.5 vol%	∼16	64
PEO/CB	Solution mixing	10 wt%	∼315	65
Epoxy/CB	Solution mixing	0.5 vol%	∼260	66
Epoxy/CB/HGB/rGO		0.2 vol%	∼600	
ENR/CB	Melt mixing	20 vol%	∼6.5 × 10^3^	67
PDMS/CB	Synthetic route	25 vol%	3.62	68
PS/PVDF/CB	Melt mixing	4.9 vol%	∼21.4	69
EPDM/PCB	Two-roll mixing	16.67 wt%/20 phr	3.3 × 10^7^	This work
		23.08 wt%/30 phr	1.4 × 10^9^	
		28.57 wt%/40 phr	2.1 × 10^9^	

a Abbreviations: AEM = ethylene acrylic elastomer, PEO = poly(ethylene oxide), HGB = hollow glass beads, rGO = reduced graphene oxide, ENR = epoxidized natural rubber, and PS = polystyrene.

**Fig. 7:**
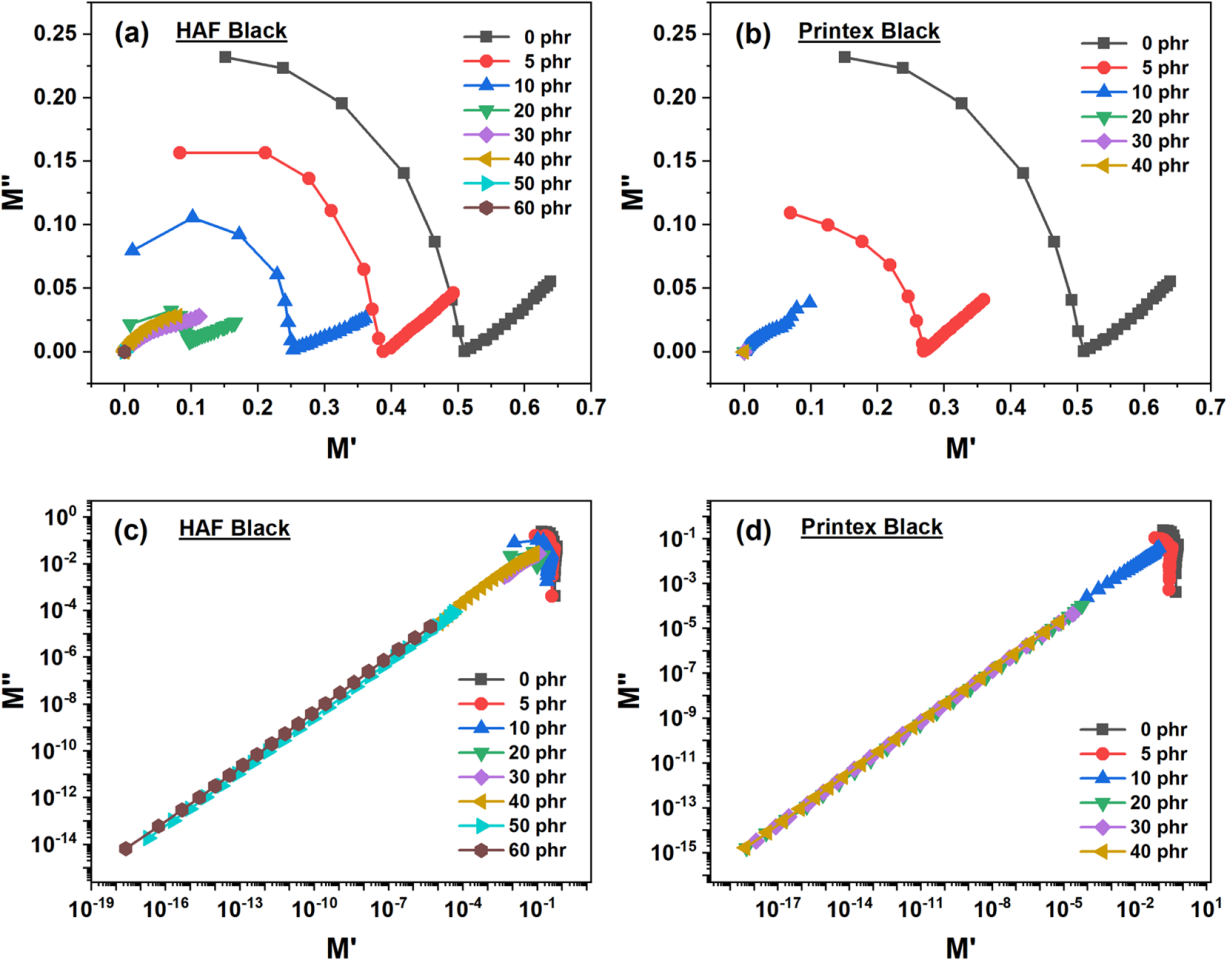
Nyquist plots of the (a) HAF and (b) Printex black filled composites on the normal scale and those of the (c) HAF and (d) Printex black filled composites on the log scale.

The dielectric polarization characteristics of the composite materials can be better understood by plotting the Nyquist plot of the samples, *i.e.*, the M′′ *vs.* M′ plots, which are given as:^70^
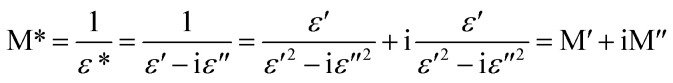
where *ε*′ and *ε*′′ are the dielectric constant and loss, respectively. The other terms, M*, M′, and M′′, are the complex, real, and imaginary parts of the electric moduli, respectively. On the basis of the above equation, we plotted M′′ *vs.* M′ for both sets of samples on normal as well as on log scales, which are shown in Fig. 7. We plotted the log scale to show the nature of the higher loaded composites, which cannot be observed on the normal scale. Actually, on the log scale, a partially semi-circle has a linear appearance. Fig. 7a and b show that the composites exhibit semicircles at a low value of M′ and the diameter of the semicircles gradually reduce with an increase in carbon loading. This is because of interfacial polarization (also known as Maxwell–Wagner–Sillars, MWS polarization) and bulk resistivity of the composites. With an increase in carbon loading, the effect of interfacial polarization increases and the bulk resistivity is reduced. As a result, the diameter of the semicircle is reduced. It can also be clearly observed from the normal scaled figures that the low-loaded composites exhibit a spike at their high value of M′. This indicates that other than interfacial polarization, other types of polarization exist. These types of polarization are known as the intermediate dipolar effect (IDE). These originate because of the movement of electrons, atoms or dipoles present in the composite systems. With an increase in filler loading, this spike is gradually reduced and diminished for higher-loaded composites. Hence, for higher-loaded composites, the MWS effect dominates the IDE effect. If we compare, it can be observed that the effect of polarization is more pronounced for Printex black filled composites compared to HAF black filled composites at equal filler loading.

#### EMI shielding effectiveness

**Fig. 8:**
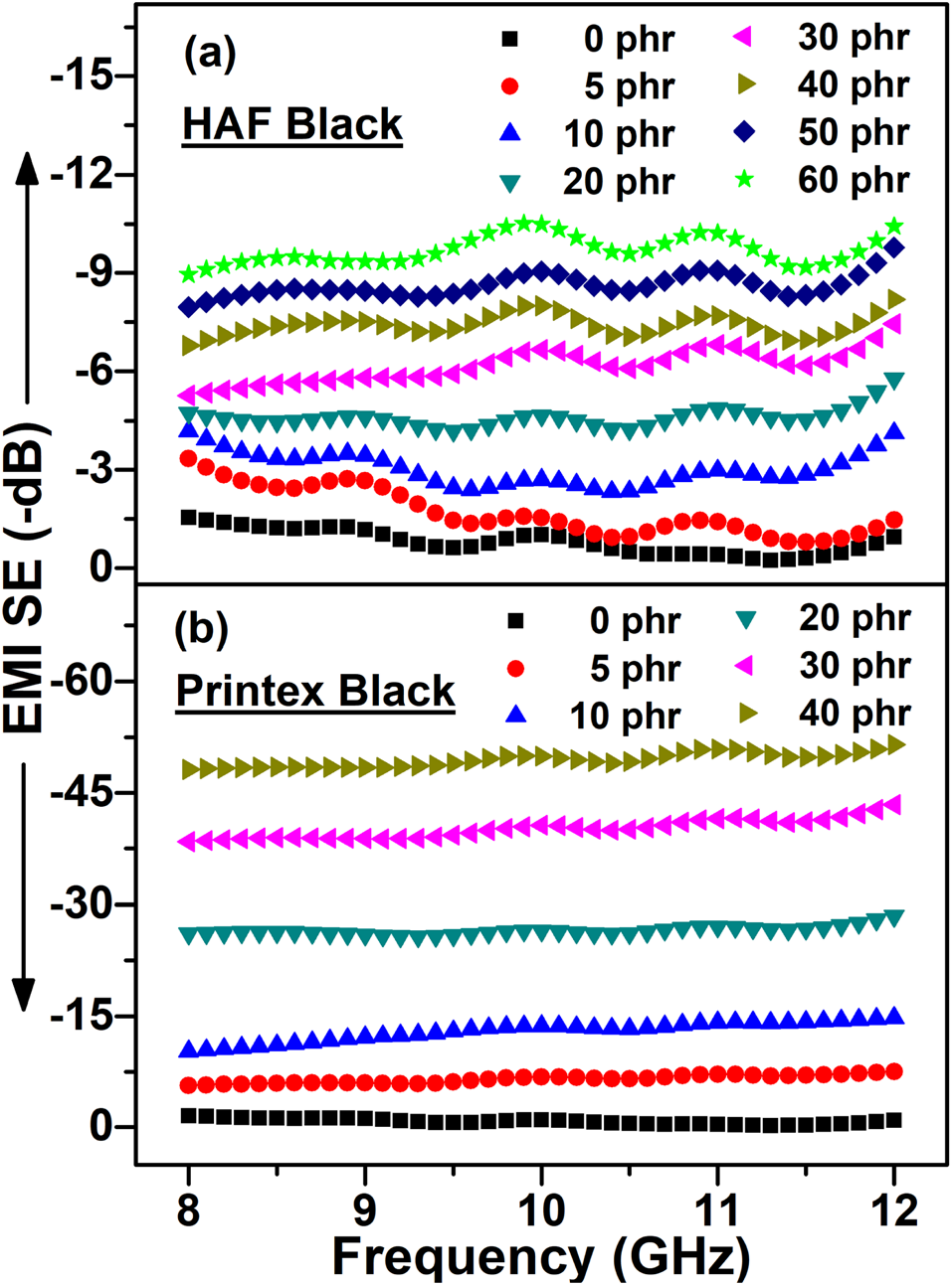
EMI SE *vs.* the frequency of the (a) HAF and (b) Printex black filled composites.

The effects of frequency (8–12 GHz) and filler loading on the EMI shielding effectiveness of the EPDM based composites filled with HAF and Printex blacks are shown is Fig. 8. It can be observed from the figure that the EMI SE fluctuates with frequency. This may be due to the irregular conductive network of the polymer composite. The figures exhibit that the EMI SE increases with an increase in filler loading for both types of carbon black filled composites. Actually, the added carbon black particles present in the composite system act as EM radiation absorption sites. With an increase in filler loading, the number of such absorption sites increases, which in turn increases the EMI SE. However, the increase in EMI SE against filler loading is more pronounced for the Printex black filled composites compared to the HAF black filled composites. Actually, the EMI SE is dependent on the conductivity, close packing, and connectivity of the conductive networks. For a high EMI SE, connectivity is more important than conductivity. Printex black gives better connectivity and conductivity in the composite compared to HAF black because of its higher structure. This is why the Printex black filled composites exhibit a higher SE compared to the HAF black filled composites. At a particular frequency 10 GHz, the EMI SE of neat EPDM, the 60 phr loaded HAF composite, and the 40 phr loaded Printex black filled composite are 1.02 dB, 10.47 dB, and 49.94 dB, respectively. Thus, there are net 9.45 dB and 48.92 dB increments in the EMI SE for the 60 phr loaded HAF and the 40 phr loaded Printex black filled composites, respectively. Hence, the EMI SE increments per phr loading are 0.1575 dB and 1.223 dB, respectively, for the 60 phr loaded HAF composite and 40 phr loaded Printex black filled composite. Thus, the increment in EMI SE per phr loading in the case of the Printex black filled composite is 7.77 times higher compared to the HAF black filled composites. As per shielding theory, 20 dB, 30 dB, 40 dB, 50 dB, 60 dB, and so on, represent attenuation blocking of 99%, 99.9%, 99.99%, 99.999%, 99.9999%, and so on, respectively.^71^ It has been mentioned in the literature that the EMI SE value of at least 20 dB is required for a material to be effectively used in EMI shielding applications.^72^ Accordingly, the HAF filled polymer composites are not effective materials for EMI shielding applications. On the contrary, the Printex black filled composites exhibit the highest EMI SE at around 50 dB. This means an attenuation of approximately 99.999%, which can be said to be a highly effective material for EMI shielding. Das *et al.* studied the EMI shielding characteristics within the X-band region of ethylene vinyl acetate (EVA) and natural rubber (NR) based composites filled with Vulcan XC-72 carbon black and short carbon fiber.^35^ It was reported that at 60 phr carbon black loading, the NR-based composite showed EMI SE values in the range of 10–12 dB, whereas the EVA-based carbon black composite showed EMI SE values in the range of 20–22 dB. These values are quite low compared to the Printex black filled composite. In an another study, three rubbers were used as base polymers mixed with Ketjen conductive carbon black and aluminum powder.^73^ These three polymers were NR, epoxidized natural rubber (ENR), and chlorosulfonated polyethylene (CSM). These carbon black filled rubber based composites showed EMI SE values in the range of 18–28 dB and the aluminum powder filled composites at around 10 dB at 50 phr loading. However, the filler 50/50 binary composites showed EMI SEs of 30 dB, 40 dB, and 35 dB for the NR, ENR, and CSM composites, respectively. The EMI shielding results of the Printex black filled composites are also higher than or comparable to some earlier reported systems loaded with 50–60 phr carbon blacks, such as nitrile rubber/carbon black composites (12 dB),^74^ EPDM/carbon black composites (8–10 dB),^75^ polyimide/carbon black composites (35–40 dB),^44^ and polyamide 6/carbon black (PA6/CB) composites (33–38 dB).^76^ Besides these, the results of this study were compared with the results of other published studies, as shown in Table 7. It can be mentioned herein that the EMI SE is dependent on polymer and filler types, preparation methods, level of filler loadings, sample thickness, *etc.* In the published literature, the composites were prepared using different techniques and the measurement thickness of the samples also varied. The value of EMI SE increases with an increase in the sample thickness. The table reveals that some hybrid composites exhibit good EMI SE values at a low filler loading. The SR/CB composite also shows good results at a low filler loading. Among the carbon black based composites, at equal sample thickness, the EMI SE result reported in this study is comparatively high. Hence, it is envisaged that the Printex black filled composites are efficient material for use in EMI shielding applications.

**Table 7:** EMI SE values of different carbon blacks and carbon black based hybrid polymer composites taken at 10.0 GHz (in some cases, approximate values are given)

Composites^a^	Filler loading (phr/wt%/vol%)	Sample thickness (mm)	Preparation method	EMI SE (dB)	Ref.
PP/CB	10 vol%	1.0	Melt blending	25.5	34
PP/PA6/CB	25 wt%	3.0	Melt compounding	26.2	52
PP/PA6/CB/SF	(25 + 25) = 50 wt%			35.1	
PS/EVA/CB	15 wt%	1.0	Melt blending, internal mixer	20.8	53
PANI/POX/CB	10 wt%	—	Solution casting	20.0	77
EMA/CB	40 wt%	5.0	Melt blending, internal mixer	30.8	38
CPE/CB	40 wt%	5.0	Melt blending, internal mixer	42.4	39
PU/CB	24 phr	2.0	Solution casting	32.4	54
PU/GN				15.8	
EMA/EOC/CB	30 wt%	2.0	Solution mixing	31.4	40
ABS/CB	3 wt%	2.0	Melt compounding	4.0	41
PVDF/CB/CI	(3 + 20) = 23 wt%	2.0	Blending and hot compaction	22.1	78
SR/CB	8 wt%	1.0	Solution mixing	20.4	55
SR/GN				13.2	
PA6,6/CB/MWCNT	(5 + 2.5) = 7.5 wt%	3.37	Melt extrusion process	10.0	56
PVDF/CB	7.5 wt%	1.0	Dry blending followed by melt mixing	11.3	57
PVDF/EG	15 wt%			11.2	
PVDF/CB/EG	(7.5 + 2) = 9.5 wt%			14.4	
PP/CB	30 wt%	40.0	Steam-chest molding	25.8	58
PP/CNT/CB	(2.5 + 2.5) = 5 wt%	2.5	Mechanical mixing	20.0	59
CPE/CNF/CB	(7.5 + 7.5) = 15 wt%	1.0	Solution blending	33.0	42
PVDF/CB/Fe_3_O_4_	(30 + 10) = 40 wt%	2.0	Solution mixing and coagulation	29.7	60
PMMA/CB	10 wt%	1.0	Solution mixing	27.6	61
PDMS/CB/CI	(17 + 40) = 57 wt%	3.0	Mechanical mixing followed by ball-milling	24.2	79
SBS/CB	CB = 15 wt%, Ag = 0.25 vol%	3.0	Solution mixing	5.06	80
SBS/CB/Ag		5.0		30.0	
UHMWPE/PP/CB	15 phr	—	Mechanical mixing	27.29	43
PI/CB	40 wt%	1.0	Solution mixing followed by hot pressing	35.0	44
SS/HDPE/CB	25 wt%	—	Melt extrusion followed by hot pressing	22.5	45
TPU/PLA/CB	30 wt%	1.0	Solution mixing	27.0	47
PDMS/CB/CNF	CB = 16.6%	0.31	Electrospinning- solution method	40.6	81
EPDM/PCB	9.09 wt%/10 phr	1.0	Two-roll mixing mill	13.7	This work
	16.67 wt%/20 phr			26.5	
	23.08 wt%/30 phr			40.6	
	28.57 wt%/40 phr			49.94	

a Abbreviations: PANI = polyaniline, POX = poloxalene, ABS = acrylonitrile butadiene styrene, and SBR = styrene butadiene rubber.

#### Morphology

**Fig. 9:**
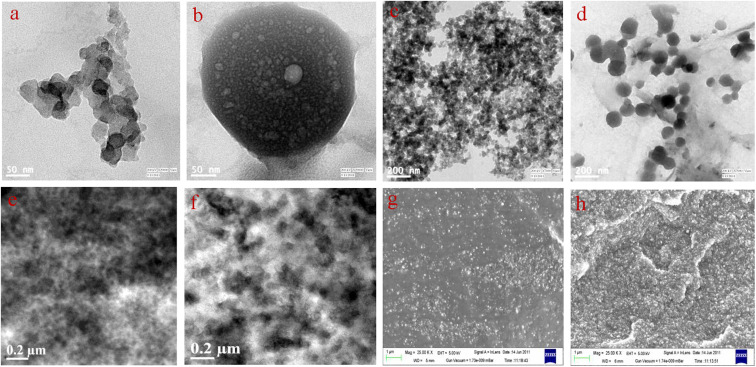
TEM images of (a) HAF black, (b) Printex black, (c) aggregates of HAF black, (d) aggregates of Printex black, (e) EPDM/HAF black composite, (f) EPDM/Printex black composite, and SEM images of (g) EPDM/HAF black composite and (h) EPDM/Printex black composite.

It was mentioned earlier that the structure of Printex black is higher than that of HAF black. To support this, the morphology of both carbon blacks and their selected composites were checked and are presented in Fig. 9. The structural difference between the two carbon blacks can be clearly observed from their transmission electron microscope (TEM) images (Fig. 9a and b). Fig. 9a shows that the particles of HAF black are fused together and form an aggregated structure. The more aggregated structure of HAF black can be observed in Fig. 9c. A single particle of Printex black is shown in Fig. 9b. Thus, on comparing, the structure of Printex black is many fold higher than that of HAF black. Fig. 9d shows that the carbon particles of Printex black are fused together to form a big aggregated structure. Hence, it can be easily understood that this higher structure of Printex black facilitates in the formation of a close compact network within the polymer matrix and thereby leads to improved electrical, dielectric, and radiation shielding properties. The TEM and FESEM images of the 40 phr loaded EPDM/HAF and EPDM/Printex composites are shown in Fig. 9e–h. The carbon particles are well distributed within the polymer matrix. Some aggregated cluster-type network structures can be observed within the polymer matrix. This facilitates the flow of charge carriers within the polymer matrix and reduces its electrical resistivity.

## Conclusions

EPDM-based polymer composites were prepared successfully using two different types of carbon blacks using a dry mixing technique and characterized to compare their effectiveness. These cured composites are environmentally sustainable. The Printex black filled composites showed superior properties compared to HAF black filled composites in all aspects of the measured characteristics. There are 4.6 times, in the order of 10^6^, and 6.65 times improvement in the tensile modulus, dielectric constant value (at 1 kHz), and EMI SE of the Printex black filled composites compared to HAF black filled composites, respectively, at their 40 phr loadings. Though the electrical percolation threshold value was determined by two different methods, both showed that the percolation threshold was approximately 1/2 to 1/3 lower for the Printex black filled composites compared to the HAF black filled composites. The AC conductivity and dielectric properties exhibited both frequency dependent and independent behavior as per the loading of carbon blacks. The Nyquist plots show that composites loaded with a low amount of filler exhibited both semicircles and spikes, indicating that interfacial as well as other types of polarization are observed for these composites. Interestingly, no spike was observed for highly loaded carbon composites. The maximum EMI SEs for the HAF and Printex black filled composites are 10.47 dB and 49.94 dB, respectively, at their highest loadings. This indicates that the HAF black filled composites are not suitable for radiation protection applications, whereas the Printex black filled composites are highly efficient. The reason behind the improvement in the abovementioned properties can be attributed to the fact that the high structure of Printex black contributes to better strength reinforcement, facilitates in the formation of a conductive network at a low loading of filler, exhibits better polymer–filler interfacial polarization, and is an excellent radiation absorber. It can be observed that the 40 phr loaded Printex black filled composite can attenuate 99.999% radiation. Thus, the composites can be used as EMI shielding materials in the aerospace and medical industries. The tensile strength for 30 phr loaded Printex black filled composites is improved by 1071% compared to base EPDM. Moreover, there are 10^7^ to 10^9^-fold increment in the dielectric constant value at 1 kHz frequency. When compared with other published results based on carbon black-polymer and hybrid filler–polymer composites, this much improvement has not previously been observed. Hence, these composites are also suitable for load bearing and supercapacitor applications. The improvement in the DC and AC conductivities of the Printex black filled composites are comparatively less than for hybrid-filler-based composites and polymer–polymer–filler ternary composites, but better than for many carbon black based composites. This can be attributed to the selective localization and preferential distribution of filler particles within the polymer matrix in the hybrid-filler-based and ternary composites. The EMI SE result in this study is comparatively higher than the values of other carbon black based composites at equal sample thickness, although some hybrid composites and SR/CB composite exhibit good EMI SE value at their low filler loadings. Finally, it can be concluded that the percolation threshold value can be determined by both methods, and a highly-conductive carbon black with a high structure is required for polymer strength reinforcement, electrical, dielectric, and radiation protection related applications.

## Author contributions

MR proposed the study, conducted the experiments, analysed the data, and wrote the entire manuscript.

## Conflicts of interest

There are no financial conflicts of interest.

## Supplementary Material

RA-013-D3RA04187E-s001
