# Supplementary material for: Superior mechanical, electrical, dielectric, and EMI shielding properties of ethylene propylene diene monomer (EPDM) based carbon black composites
Source: RSC Adv. 2023 Aug 24;13(36):25443–58. doi: 10.1039/d3ra04187e (PMC10448605; doi:10.1039/d3ra04187e)
Supplement: RA-013-D3RA04187E-s001 [file RA-013-D3RA04187E-s001.pdf]

Electronic Supplementary Material (ESI) for RSC Advances.
This journal is © The Royal Society of Chemistry 2023

Superior mechanical, electrical, dielectric, and EMI shielding properties of ethylene propylene diene monomer (EPDM) based carbon blacks' composites

Mostafizur Rahaman

Department of Chemistry, College of Science, King Saud University, P.O. Box 2455, Riyadh 11451, Saudi Arabia

Corresponding author's email: mrahaman@ksu.edu.sa (MR)

Table S1. General specification of HAF and Printex XE2 carbon blacks.

Typical properties	HAF Black	Printex Black	Unit
Mean particle size	31	35	nm
Surface area, STSA	75	587	m^2/g
Surface area, CTAB	78	600	m^2/g
DBP absorption	88-102	350–410	cc/100 g
Volatiles at 105 °C	1.5	1.0	%
